# Supplementary figures and images for: Activity of MukBEF for chromosome management in E. coli and its inhibition by MatP
Source: eLife. 2024 Feb 5;12:RP91185. doi: 10.7554/eLife.91185 (PMC10945525; doi:10.7554/eLife.91185)

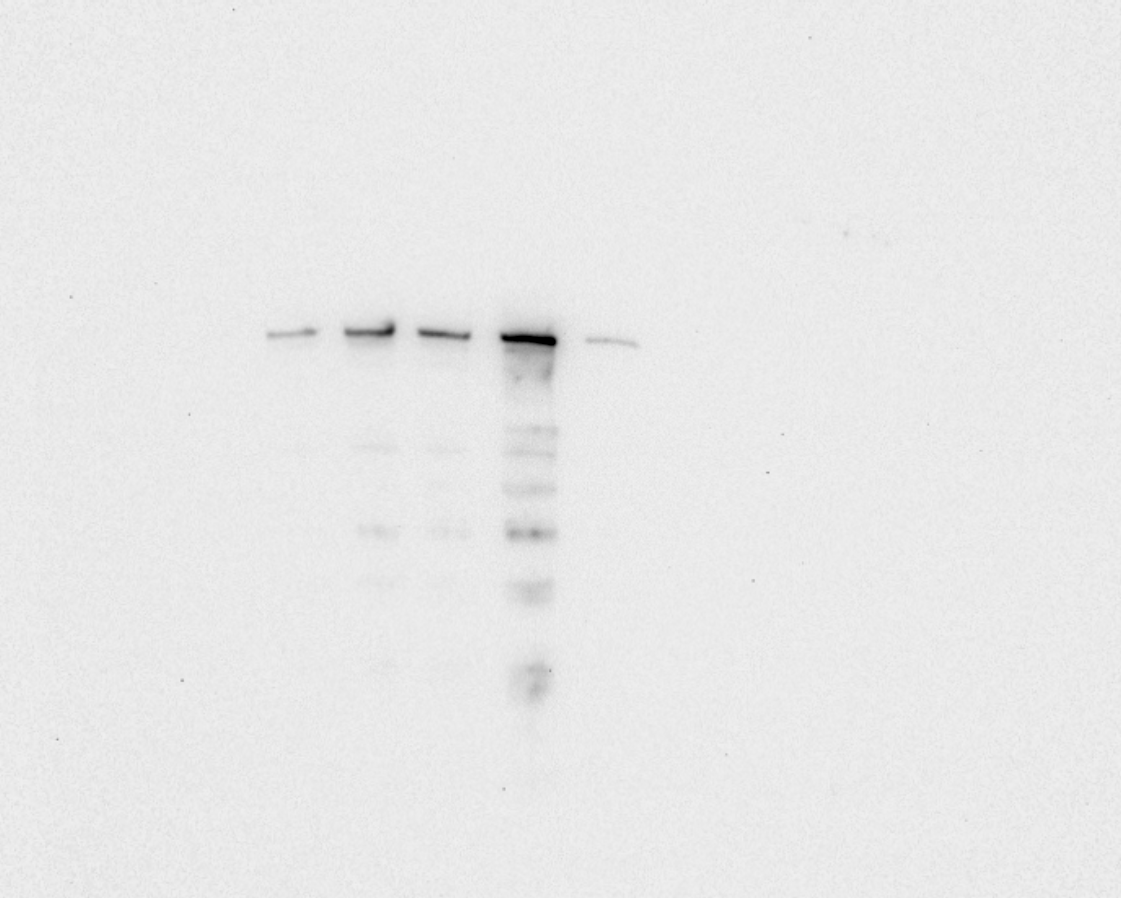

Supplement: Figure 1—figure supplement 1—source data 1. [file elife-91185-fig1-figsupp1-data1.zip › Figure 1-figure_supplement1_source data1.tif]

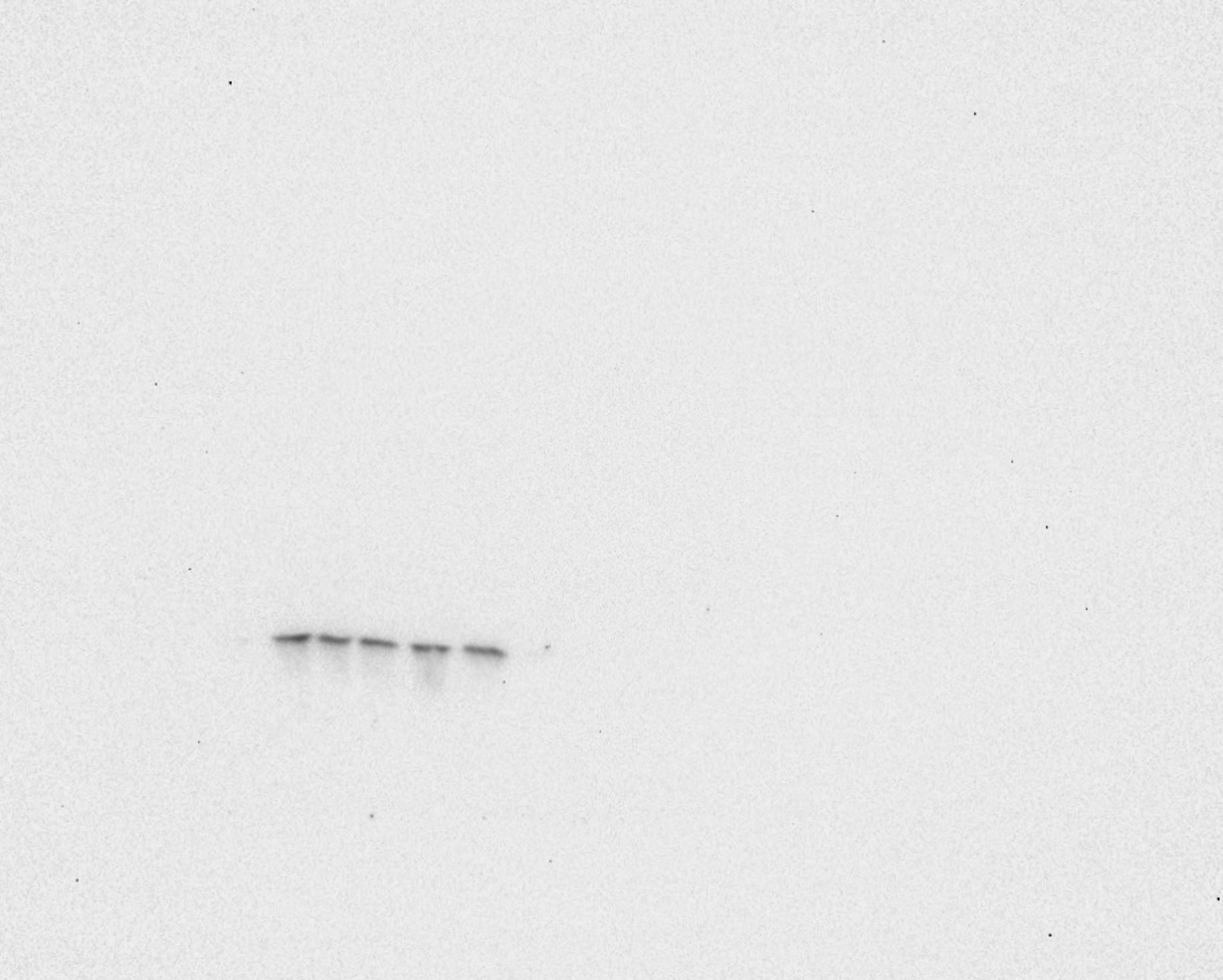

Supplement: Figure 1—figure supplement 1—source data 2. [file elife-91185-fig1-figsupp1-data2.zip › Figure 1-figure_supplement1_source data2.tif]
