# Supplementary material for: Activity of MukBEF for chromosome management in E. coli and its inhibition by MatP
Source: eLife. 2024 Feb 5;12:RP91185. doi: 10.7554/eLife.91185 (PMC10945525; doi:10.7554/eLife.91185)
Supplement: Figure 1—figure supplement 1—source data 3. [file elife-91185-fig1-figsupp1-data3.zip › Figure 1-figure_supplement1_source data3.pdf]

Western blot analysis of MukB-flag expression

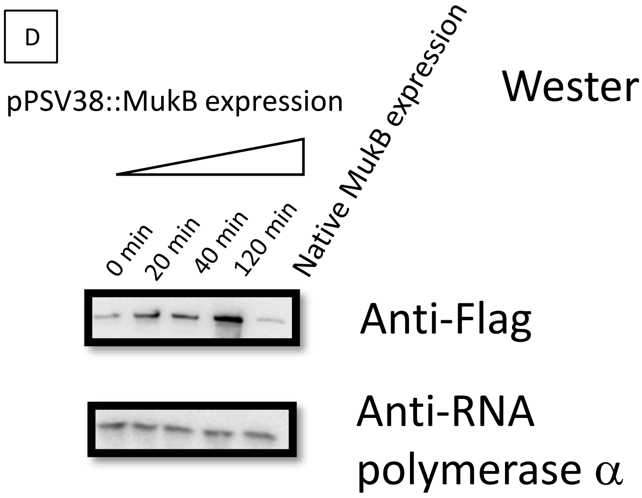

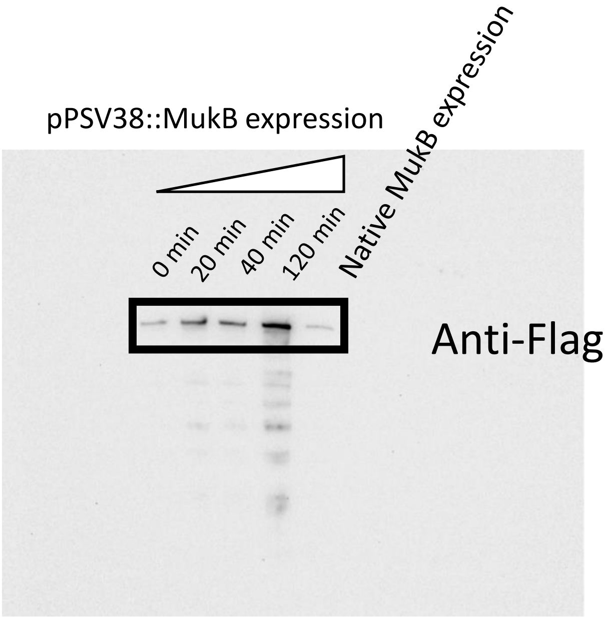

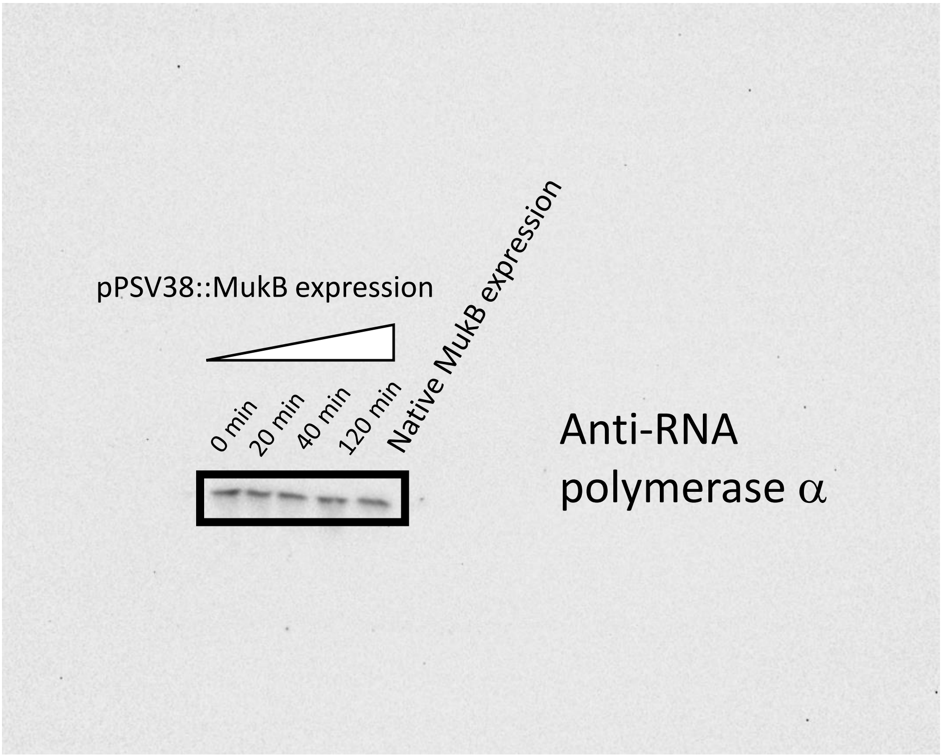

Figure 1- figure supplement 1

Immunoblot analysis showing MukB-Flag accumulation in the *ΔmukF* pPSV38::mukFEB-Flag strain during the induction time course. IPTG was added for 0, 20, 40, and 120 minutes, as in Figure 1 (first 4 lanes on the left), before immunoblotting. The same immunoblot was conducted on exponentially growing cells expressing MukB-Flag from the natural *mukBEF* promoter on MM at 22°C (under the same growth conditions as in Figure 1). Loading control was performed using the quantification of the α subunit of the RNA polymerase (detected with the commercially available Biolegend antibody). Full complementation was observed in Figure 1 after 120 minutes of induction, corresponding to a tenfold increase in expression.
