# Supplementary material for: Activity of MukBEF for chromosome management in E. coli and its inhibition by MatP
Source: eLife. 2024 Feb 5;12:RP91185. doi: 10.7554/eLife.91185 (PMC10945525; doi:10.7554/eLife.91185)
Supplement: Supplementary file 1. [file elife-91185-supp1.docx]

| **Strain name** | **Description** | | **Macrodomaines coordinates** | | **Reference** |
| --- | --- | --- | --- | --- | --- |
| *WT (before transposition)* | *MG1657 (MG1655 ΔlacIZ phi80^+^ΔattB::aadA)* | | Ori(3.7Mb-4.6Mb)-NSR(0-0.6Mb)-Right(0.6-1.2Mb)-Ter(1.2-2Mb)-Left(2-2.8Mb)-NSL(2.8-3.7Mb) | | (Valens et al., 2004) |
|  | **Transposition coordinates** | | | **Configuration after transposition** |  |
|  | ***attL*** | ***attR*** | ***attB*** |  |  |
| *RiTer11* | 1099533 | 651775 | 1532480 | Ori-NSR-Right(152Kb)-Ter2(330Kb)-Right(447 Kb)-Ter1(470Kb)-Left-NSL | This work |
|  | ***attL*** | ***attR*** | ***attB*** |  |  |
| *LiTer15* | 3250851 | 2202961 | 1596000 | Ori-NSR-Right-Ter1(390Kb)-Left(1Mb)-Ter2(410Kb)-Left(202Kb)-NSL(449Kb) | This work |
| *LiTer9* |  |  | 1710441 | Ori-NSR-Right-Ter1(501Kb)-Left(1Mb)-Ter2(299Kb)-Left(202Kb)-NSL(449Kb) | This work |
| *LiTer7* |  |  | 1746766 | Ori-NSR-Right-Ter1(550Kb)-Left(1Mb)-Ter2(250Kb)-Left(202Kb)-NSL(449Kb) | This work |
| *LiTer4* |  |  | 1814000 | Ori-NSR-Right-Ter1(610Kb)-Left(1Mb)-Ter2(190Kb)-Left(202Kb)-NSL(449Kb) | This work |
| *LiTer4 ΔmatS28* |  |  |  | Ori-NSR-Right-Ter1(610Kb)-Left(1Mb)-Ter2(133Kb)-Left(259Kb)-NSL(449Kb) | This work |

MG1655 (*F- lambda- ilvG- rfb-50 rph-1*) lab collection

MG1657 (MG1655 *ΔlacIZ phi80^+^ΔattB::aadA*) (Valens et al., 2004)

MG1655 *dnaC2 tet* *mukB-Spa-TAG* *cm*  this work

MG1655 *dnaC2 tet mukB-Spa-TAG apr* this work

MG1655 *dnaC2 tet mukB-Spa-TAG apr ΔmukF::cm* this work

MG1655 *matP-spa-TAG cm* (Dupaigne et al., 2012)

MG1655 *matPC20-spa-TAG cm* this work

MG1655 *matP-spa-TAG ΔmatS9::kan-10::zeo-11::cm-12::apr* this work

MG1657 RiTer11 *attL cm* 1099533 *attR kan* 651775 *attB apr* 1532480 this work

MG1657 RiTer11 *ΔmatP::zeo* this work

MG1657 RiTer11 *ΔmukB::zeo* this work

MG1657 LiTer15 *attL cm* 3250851 *attR kan* 2202961 *attB apr* 159600 this work

MG1657 LiTer15 *ΔmukB::apr* this work

MG1657 LiTer9 *attL cm* 3250851 *attR kan* 2202961 *attB apr* 1710441 this work

MG1657 LiTer7 *attL cm* 3250851 *attR kan* 2202961 *attB apr* 1746766 this work

MG1657 LiTer7 *ΔmatP::zeo* this work

MG1657 LiTer7 *dnaC2 tet MukB-Spa-TAG cm* this work

MG1657 LiTer4 *attL cm* 3250851 *attR kan* 2202961 *attB apr* 1814000 this work

MG1657 LiTer4 *ΔmatS26::zeo* this work

MG1657 LiTer4 *ΔmatS28::apr* this work

MG1657 LiTer4 *ΔmatP::zeo* this work

MG1655 *matP5A apr* this work

MG1655 *ΔmukF::cm* this work

MG1655 *ΔmukB::apr* this work

MG1655 *ΔmukF::cm ppSV::empty* this work

MG1655 *ΔmukF::cm ppSV::mukFEB* this work

MG1655 *ΔmukF::cm ppSV::mukFEB-spa-TAG* this work

| **Strain name** | ***matS sites* positions** | | | |
| --- | --- | --- | --- | --- |
|  | ***matS1*** | | ***matS28*** | |
| ***WT*** | 1136538 | | 1974386 | |
| ***RiTer11*** | **Ter1** | | **Ter2** | |
|  | ***matS1*** | ***matS11*** | ***matS12*** | ***matS28*** |
|  | 1136538 bp | 1067196 bp | 1536807 bp | 1974386bp |
| ***LiTer15*** | ***matS1*** | ***matS13*** | ***matS14*** | ***matS28*** |
|  | 1136538 bp | 1563282 bp | 2648362 bp | 3022276 bp |
| ***LiTer9*** | ***matS1*** | ***matS19*** | ***matS20*** | ***matS28*** |
|  | 1136538 bp | 1704014 bp | 2769807 bp | 3022276 bp |
| ***LiTer7*** | ***matS1*** | ***matS21*** | ***matS22*** | ***matS28*** |
|  | 1136538 bp | 1745151 bp | 2813103 bp | 3022276 bp |
| ***LiTer4*** | ***matS1*** | ***matS24*** | ***matS25*** | ***matS28*** |
|  | 1136538 bp | 1810136 bp | 2886716 bp | 3022276 bp |
| ***LiTer4 ΔmatS28*** | ***matS1*** | ***matS24*** | ***matS25*** | ***matS27*** |
|  | 1136538 bp | 1810136 bp | 2886716 bp | 2964754 bp |
